# Benign Paroxysmal Positional Vertigo (BPPV): History, Pathophysiology, Office Treatment and Future Directions

**DOI:** 10.1155/2011/835671

**Published:** 2011-07-25

**Authors:** Jeremy Hornibrook

**Affiliations:** Department of Otolaryngology, Head and Neck Surgery, Christchurch Hospital, 2 Riccarton Avenue, Christchurch 8011, New Zealand

## Abstract

BPPV is the most common cause of vertigo. It most often occurs spontaneously in the 50 to 70 year age group. In younger individuals it is the commonest cause of vertigo following head injury. There is a wide spectrum of severity from inconsistent positional vertigo to continuous vertigo provoked by any head movement. It is likely to be a cause of falls and other morbidity in the elderly. Misdiagnosis can result in unnecessary tests. The cardinal features and a diagnostic test were clarified in 1952 by Dix and Hallpike. Subsequently, it has been established that the symptoms are attributable to detached otoconia in any of the semicircular canals. BPPV symptoms can resolve spontaneously but can last for days, weeks, months, and years. Unusual patterns of nystagmus and nonrepsonse to treatment may suggest central pathology. Diagnostic strategies and the simplest “office” treatment techniques are described. Future directions for research are discussed.

## 1. History and Pathophysiology

Benign paroxysmal positional vertigo (BPPV) is the most common vertiginous disorder in the community. The cardinal symptom is sudden vertigo induced by a change in head position: turning over in bed, lying down in bed (or at the dentist or hairdresser), looking up, stooping, or any sudden change in head position. There is a wide spectrum of severity. Mild symptoms are inconsistent positional vertigo. Moderate symptoms are frequent positional attacks with disequilibrium between. When severe, vertigo is provoked by most head movements, giving an impression of continuous vertigo. The symptoms can last for days, weeks, months, or years, or be recurrent over many years.

The earliest reference to it may have been by Shakespeare in “Romeo and Juliet” [1] In Act I, Scene II [enter Romeo and Bevolio] Bevolio says “Tut man, one fire burns out another's burning. One pain is lessen'd by another's anguish; turn giddy, and be holp by backwards turning….” In the medical literature the first descriptions of positionally induced vertigo are attributed to Adler [2] and later Barany [3], who believed it was a disorder of the otolith organs. Barany elicited vertigo in a 27-year-old woman by turning her head from side to side in a supine position and noted “…there appeared a strong rotatory nystagmus to the right with a vertical component upwards, which when looking to the right was purely rotatory, and when looking to the left was purely vertical.” In 1952 Margaret Dix (1911–1981) and Charles Hallpike (1900–1979) [4] at Queen Square Hospital, based on 100 patients, presented a symptomatological definition and a provocative positional test for what they called “positional nystagmus of the benign positional type.” For symptoms they note: “The story given by the patient is characteristically that the giddiness comes on when he lies down in bed or when he turns over in bed, or when such a position is taken up during the day; for instance lying down beneath a car or in throwing the head backward to paint a ceiling.” Their diagnostic test: “….the patient is first seated upon the couch with the head turned to one side and the gaze fixed firmly on the examiner's forehead. The examiner then grasps the patient's forehead firmly between his hands and briskly pushes the patient back into the critical position [30 degrees below the level of the couch and turned some 30 to 45 degrees to one side]. The reaction which results calls for some detailed description.” As did Barany they noted a torsional nystagmus with the upper pole of the eye beating (fast phase) toward the ground and that it “fatigued” on retesting. Additionally, they observed a response latency of approximately 5 seconds, a crescendo and decline of nystagmus, and a reversal of the nystagmus as the patient sits up. To eliminate the possibility that the response could be induced by vascular occlusion from rotation of the neck they tested patients on an apparatus which avoided it. The same response occurred. In Britain Hallpike was a pioneer of temporal bone histology. The right temporal bone of 40-year-old woman with “positional nystagmus of the benign positional type…to the right with the right ear undermost” was examined. In the macula of the utricle, the otolithic membrane was absent. They concluded: “The general picture is one of chronic tissue changes resulting either from infection or trauma…” and “We are thus directed to the conclusion that the lesion *is* a peripheral one and in the labyrinth towards which, when undermost, the nystagmus is directed”. Hallpike provided further evidence for a peripheral cause by abolishing symptoms in two patients with a chemical labyrinthectomy of an acoustically dead ear [5] and in one patient by an eight nerve section [6]. Both Barany and Dix and Hallpike concluded that “positional nystagmus of the benign positional type” was caused by disorder of the utricular macula.

By the early 19th century the bony and some membranous structure of the inner ear were anatomically well described but their functions unproven. Common notions were the following. The cochlea was responsible for mediating the nature and pitch of sound; the saccule and utricle were for perception of loudness, and the semicircular canals for transmission of bone-conducted sound and perception of sound direction [7]. Marie-Jean Flourens (1794–1867) was a professor of comparative anatomy in Paris, and in 1824 he published his experimental results on pigeon semicircular canals [8]: “If the membranous ducts are injured, a painful sensitivity to tones is observed, accompanied by abrupt and violent movements of the head…. If the horizontal canals are severed, the animal turns on its vertical axis; if the posterior vertical canal is severed the animal rolls over backward, and if the anterior vertical canal is severed the animal falls forward….” Flourens concluded that the semicircular canals inhibited motion (“forces moderatrices”) and influenced direction of motion, rather than having a role in balance. Flourens' work had been largely ignored but was known to Prosper Meniere and acknowledged in his final paper in 1861 [9]. According to Adam Politzer (1835–1920) in his “History of Otology” [10] “the realization that the vestibular and semicircular canal structures are not organs of sound perception, that sound perception is transmitted solely through the cochlea, is the single most important result of Flourens' experiments”. However it was another sixty years until a more sophisticated understanding of semicircular canal functions and their generated nystagmus was achieved by Julius Ewald (1855–1921) who was later Professor of Physiology at the University of Strassburg (now Strasbourg). In pigeons, he cannulated each semicircular canal and applied negative and positive pressures and observed the directions and intensity of the induced nystagmus [11]. The two major findings have become known as Ewalds' Laws: (1) the direction of the induced nystagmus is in the plane of the canal being stimulated, and (2) in the horizontal canal an ampullopetal (towards the vestibule) movement of endolymph causes the greatest response where as in the posterior and superior canals an ampullofugal (away from the vestibule) endolymph movement causes the greatest response. At the time the differences were perplexing, as expressed by a writer in 1920 [12]: “It is, however, difficult to imagine how the same endolymph current can be stimulating for the one endorgan and hindering for the other”. 

**Figure 1:**
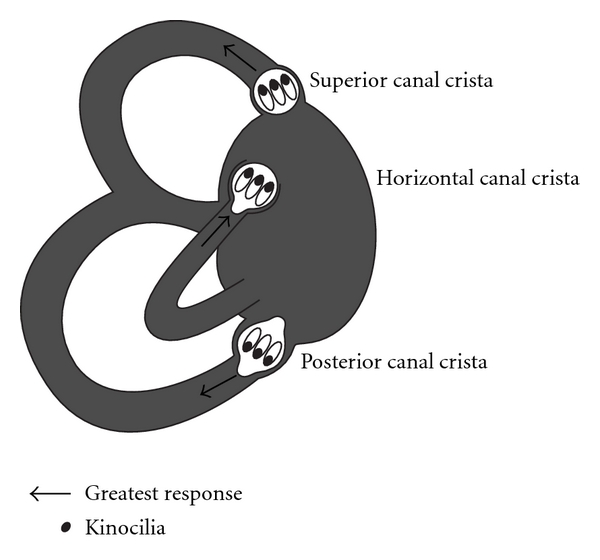
Orientation of kinocilia in the semicircular canal cristae. In the horizontal canal kinocilia are on the vestibule side. In the posterior and superior canals kinocilia are on the canal side.

**Figure 2:**
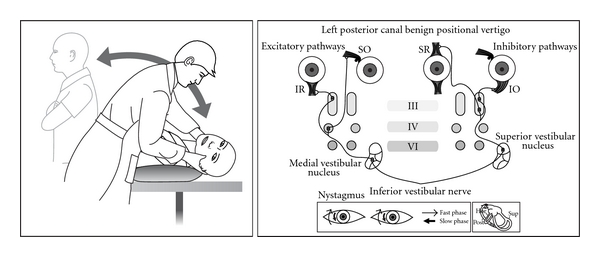
Posterior canal BPPV in a left ear showing Dix Hallpike test, inner ear, and receptor connections to the extraocular muscles.

**Figure 3:**
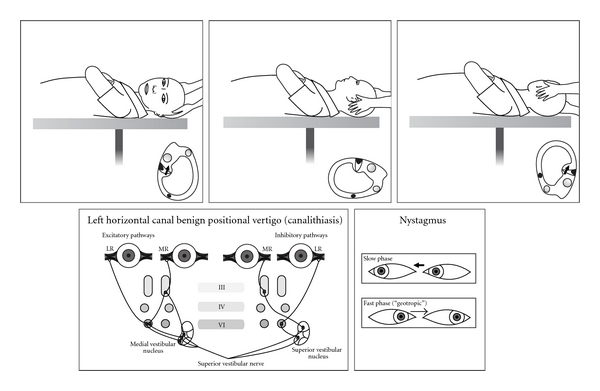
Horizontal canal BPPV (canalithiasis) in a left ear showing Head Roll test, inner ear, and receptor connections to the extraocular muscles.

**Figure 4:**
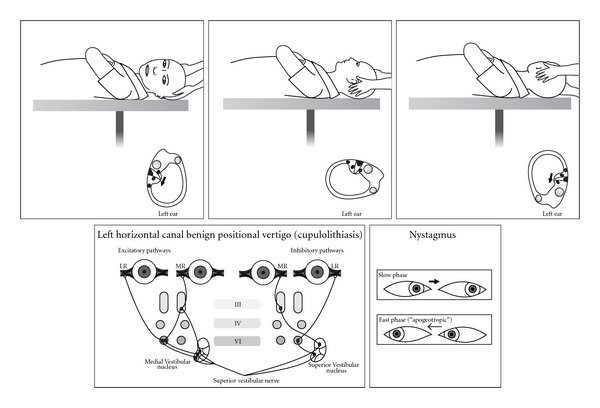
Horizontal canal BPPV (cupulolithiasis) in a left ear showing Head Roll test, inner ear, and receptor connections to the extraocular muscles.

**Figure 5:**
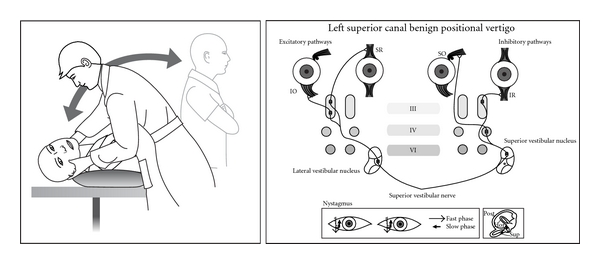
Superior canal BPV in a left ear showing Dix Hallpike test, inner ear, and receptor connections to the extraocular muscles.

Thirty years later the advent of the electron microscope allowed a more detailed view of inner ear ultrastructure. In 1954 Wersall [13] showed that each vestibular sensory cell has one kinocilium and many stereocilia. The finding of morphological polarization of kinocilia on vestibular sensor cells [14, 15] explained Ewald's paradox. In horizontal canal cristae the kinocilium is on the vestibule side of the stereocilia; in the posterior and superior canals the kinocilium is on the canal side of the stereocilia (Figure 1). In the 1960s, experiments in cats [16] clarified the relationship between canal receptors and extraocular muscles. Each receptor is connected to one ipsilateral and one contralateral muscle. The second order neurones are either excitatory (to the agonist muscles) or inhibitory (to the antagonist muscles) (Figures 2, 3, 4, and 5).

**Figure 6:**
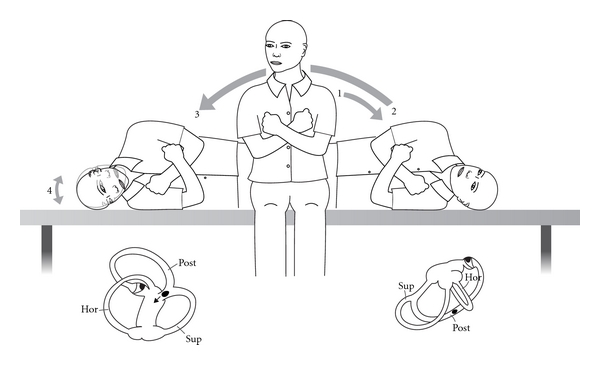
Semont maneuver for posterior canal BPPV in a left ear.

In 1962 Harold Schuknecht (1917–1996) at Harvard University in Boston [17] proposed that BPPV “might be caused by detached utricular otoconia, acting upon the cupula of the posterior semicircular canal. Although at that time there were no confirming human pathological studies, the concept seemed plausible from a purely theoretical point of view.” In 1969 Schuknecht [18, 19] confirmed finding basophilic staining masses attached to the posterior canal cupula in patients who had had BPPV symptoms. He called this cupulolithiasis (heavy cupula) and assumed the masses were detached utricular otoliths which were removed by decalcification in preparation. This was supported by Gacek's report of five patients where the selective resection of the posterior ampullary nerve abolished BPPV symptoms [20]. Cupulothiasis became the dominant theory for nearly thirty years, although it did not explain the variable and often long latency and fatiguability of the nystagmus. It was the impetus for two early specific treatments. Previously “treatment” had been by Cawthorne's exercises in which the patient was instructed to repeat continually any movement which caused the vertigo until it ceased, on the assumption that central adaption was occurring [21]. Based on the cupulolithiasis theory Brandt and Daroff [22] devised an inpatient treatment where subjects lay down to the provocative side, sat up for thirty seconds, and then lay to the other side every three hours. After seven to ten days, 61 of 67 subjects were free of symptoms. The assumed aim was detachment of the particle from the posterior canal cupula. In France Semont (a physiotherapist) and Sterkers [23, 24] modified this to a logical physician-controlled treatment they called the Liberatory maneuver, now known as the Semont maneuver (Figure 6). The patient is lain down to the side of the symptomatic ear, facing down. When the nystagmus ceases, the patient is moved rapidly through 90 degrees to the opposite side (where the symptomatic ear becomes uppermost). Either immediately or up to 15 seconds later the patient experiences vertigo and has nystagmus identical to the symptomatic side. The technique was little known outside France.

**Figure 7:**
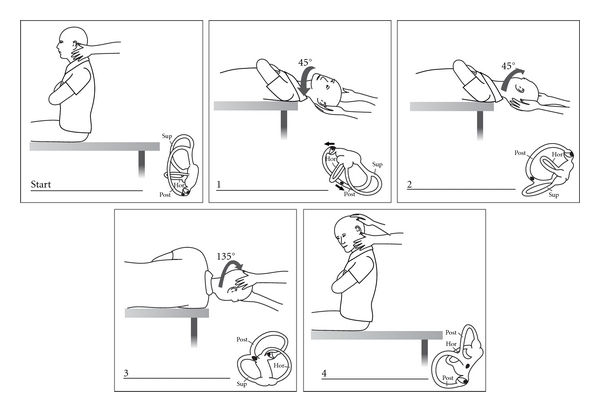
Epley canalith repositioning procedure (CRP).

In attempting to explain the latency and fatiguability of BPPV nystagmus, Hall et al. [25] (at the University of London, Ontario) and later Epley [26] (a solo private practice otologist in Portland, Oregon) made models of the semicircular canals and proposed that they were better explained by free-floating particles in the posterior canal, which Epley called canalithiasis. Also at the University of London, Ontario, Parnes and McClure, in attempting a surgical posterior canal occlusion, observed and photographed free otoconia in the endolymphatic compartment [27]. Based on his models Epley proposed a controlled set of head movements he called the canalith repositioning procedure (CRP) [28] (Figure 7). Epley had presented this as an instruction course at the American Academy of Otolaryngology, Head and Neck Surgery meetings since 1980 and endured considerable derision because he used a heavy massage vibrator over the mastoid process [29]. After seeing canaliths at operation, Parnes [30] described an almost identical particle repositioning maneuver (PRM) (often known as the Modified Epley maneuver) whose main difference is its slower pace.

BPPV (85% posterior canal) is now recognized as the most common cause of vertigo in adults. It is estimated that 2.4% of people experience at least episode in their life [31]. 9% of residents in a home for the elderly were found to have BBPV [32]. The onset is most commonly between the fifth and seventh decades. It is the most common cause of vertigo after a head injury [33, 34]. An episode of vestibular neuritis [35] and a period of bed rest [36] are common antecedents. Omission of a simple clinical test can result in patients undergoing unnecessary, expensive investigations [37]. 

Previously “nontypical” forms of positionally induced nystagmus were assumed to always have a central cause. While performing CRPS, Epley observed a sudden change of “typical” torsional posterior canal nystagmus to horizontal direction-changing nystagmus and deduced the nystagmus that would be caused by otoconia in the horizontal and even the superior canal [38]. Without clinical proof Epley predicted the logical treatment for horizontal canal BPPV would be a 360 degree horizontal plane rotation away from the symptomatic ear. In 1985 McClure [39] had published the electronystagmographic (ENG) traces of seven subjects who had intense positional vertigo and direction-changing horizontal nystagmus when supine. The fast phase was towards the undermost ear (geotropic). McClure suspected a “viscous plug” in the horizontal canal which was causing a piston effect on the horizontal canal receptor. As discovered by Ewald, an ampullopetal (towards the vestibule) cupula deflection is known to cause the most intense nystagmus and vertigo. Horizontal canal BPPV was then reported by others [40–43] and its particularly intense vertigo confirmed. Early repositioning attempts failed [41]. A 270 degree “barbecue” rotation was trialled [44].

These simple horizontal repositioning techniques remain the usual way of treating the horizontal variant of BPPV (Figure 10). Occasionally the most intense nystagmus is away (apogeotropic) from the undermost ear, implying a particle or particles attached to the cupula, or close to it, on its canal or utricular side [43, 45–47]. The cupula becomes “heavy” and is ampullofugal when the symptomatic ear is undermost and ampullopetal when it is uppermost. It can be difficult to ascertain which is the symptomatic ear, but it is likely to be the *undermost ear *which initiates the *least nystagmus * (Figure 4). Horizontal canal BPPV comprises approximately 15% in most series. As for posterior canal it can occur *de novo*, after mild head injury or by “canal conversion” during posterior canal repositioning [45, 46]. It is likely that patients with horizontal canal BPPV inadvertently treat themselves by rolling over in their sleep, if it is in the desirable direction. It they turn in the “wrong” direction they trigger and awake with vertigo.

Although Brandt et al. [48] in 1994 had alluded to “the rare anterior [superior] canal BPPV, the spontaneous symptoms occur when the affected ear is uppermost”, the first detailed description of superior canal BPPV is usually attributed to Herdman and Tusa [49] who documented two patients whose positionally induced nystagmus was accompanied by downbeat and torsional nystagmus likely to be caused by a superior canal receptor and which ceased after repositioning treatment, implying it was rare form of BPPV. Subsequently superior canal BPPV was recognized and reported by others [50–56] in whose series it accounts for approximately 1% of all BPPV diagnoses. In a review [52] of 50 consecutive patients with positionally induced nystagmus, 75% had a central cause: multiple system atrophy, cerebellar degeneration, and other miscellaneous causes with immediate onset of downbeat nystagmus on a Dix Hallpike test. In 25% (“idiopathic”) a Dix Hallpike test or a head-hanging test elicited downbeat nystagmus with a short latency. In half the subjects a torsional nystagmus could be seen through Frenzel glasses, but in one it was only discernible by video imaging. Aw et al. [54] studied forty-four patients whose BPPV had not responded to conventional repositioning, using 3-dimensional research coils and a 2-axis whole-body rotator. Seven had downbeat nystagmus with a small torsional component, and all responded to a “head-over-heels” forward rotation in the plane of the superior canal. Differences in the ampullary segments of the posterior and superior canals most likely explain why superior canal BPPV downbeat nystagmus can be triggered by a Dix Hallpike test to either side and for its small (or absent) torsional component. In most cases the symptomatic ear is the *uppermost ear* (Figure 5).

## 2. Office Management

The author uses the simplest repositioning techniques.

### 2.1. Posterior Canal BPPV

Diagnosis is by the Dix Hallpike test. Older patients with neck, back, and hip problems require special care, and the test can be more simply done over a pillow (Figures 2 and 5). The patient MUST experience vertigo. Occasionally an initial negative test may become strongly positive after the patient does vigorous headshaking.

**Figure 8:**
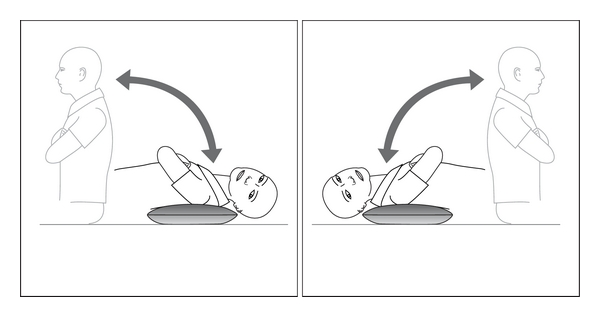
Self-testing at home by lying down over a cushion.

The most common repositioning treatment is Epley's CRP as modified by Parnes with one-minute pauses between head positions (Figure 7). Following the CRP a repeat Dix Hallpike test is done. If positive, the CRP is repeated with mastoid vibration (Hitachi Magic Wand 250 Hz massage vibrator). If the test is negative, no further repositioning is done. However it is NOT confirmation of treatment success, and retesting should be done. There is no widely accepted interval. One week is a reasonable goal. Younger, agile patients can be shown how to conduct their own follow-up test at home by lying down over a cushion on the floor (Figure 8). However, older patients, who often report success by avoiding provocative positions, MUST be seen and formally retested. 

**Figure 9:**
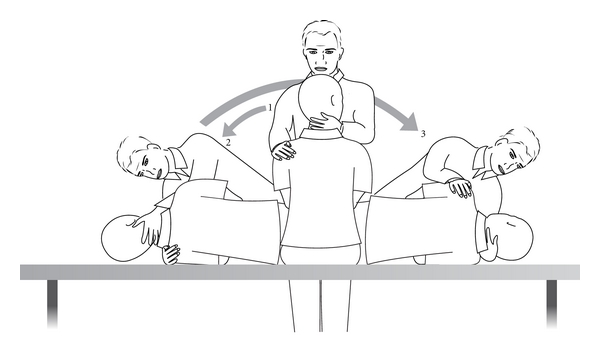
Safer technique for performing the Semont maneuver, with the patient moved by a hand under the shoulder and the other hand supporting the neck.

If at followup the Dix Hallpike test is positive, repeat treatment can be by a further CRP with mastoid vibration or by the Semont maneuver. Descriptions of the Semont maneuver typically show the patient held and moved by two hands around the neck. This is extremely inappropriate for many individuals who are larger, obese, or who have neck problems. A safer technique is for the physician to rapidly move the patient from side to side by a hand under the downmost shoulder (symptomatic ear) and the other supporting the neck (Figure 9).

A Cochrane collaboration review of the Epley CRP and 5 subsequent random controlled trials found a significant success compared with nontreated controls [57, 58]. Comparison of trials is confounded by variation in the number of CRP cycles used per treatment, clinician experience, and the treatment setting. There have been relatively few trials [59, 60] confirming the efficacy of the Semont maneuver compared with sham treatment. The Semont maneuver is the most logical first treatment for a patient with posterior canal cupulithiasis (immediate nystagmus onset) with attached otoconia more likely to be dislodged by a centrifugal force.

Epley initially recommended that after a CRP the patient should sleep propped up for two nights to prevent repositioned particles from returning [28]. However, numerous studies have not shown any advantage from posttreatment restrictions [61, 62]. The use of adjunctive mastoid vibration has remained contentious, probably because of the power range of the devices used [63].

**Figure 10:**
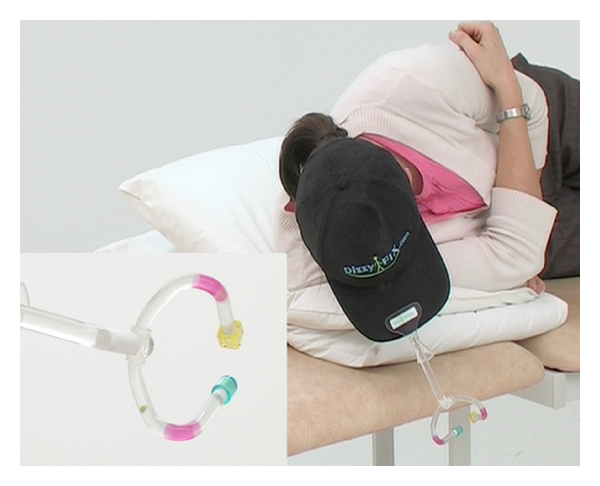
“DizzyFix” dynamic visual device for teaching the modified Epley CRP to patients and health professionals. It is not a model of the semicircular canals but a representation assisting accurate head positioning and appropriate timing for a posterior canal particle to be successfully expelled.

The vast majority of BPPV treatment studies have been performed in specialist practice settings. While very few patients can or even wish to administer self-treatment, it is an understandable goal. Self-administered CRP at home after initial office CRP achieved a slightly greater improvement [63]. As an adjunct to self-treatment the newly released “DizzyFix” dynamic visual device (Clearwater Clinical) [64] significantly improved the performance of volunteers learning Parnes' modification of the CRP (Figure 10). It is a useful teaching tool on correct CRP technique for patients and health professionals.

### 2.2. Horizontal Canal BPPV

**Figure 11:**
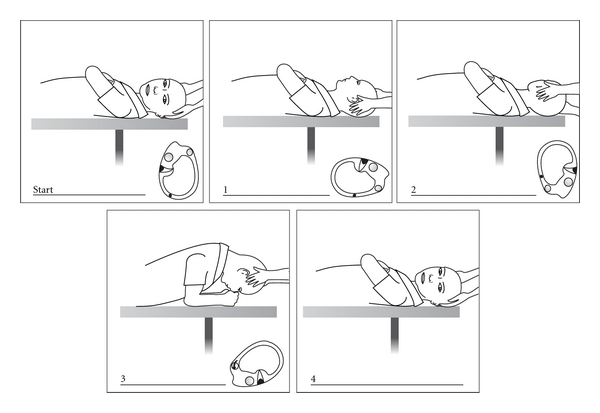
“Barbecue” repositioning for horizontal canal BPPV in a left ear.

Horizontal canal BPV is most likely to be discovered as the patient is undergoing a Dix Hallpike provocative test [46, 47]. Occasionally it suddenly becomes apparent (“canal Conversion”) after a CRP, with brisk horizontal-rotatory nystagmus. If there is pillow under the shoulders, it is removed and the patient is moved down the examination couch so that the head is midline and in the horizontal plane. Then the head is gently turned to one side and then the other (“head roll” test). Usually there is a clear, repeatable pattern of brisk nystagmus towards one undermost ear (maximum geotropic) and then weaker nystagmus (apogeotropic) when the opposite ear is down. If a “canal conversion” has occurred, the symptomatic ear is already known. The vertigo tends to be more intense than for posterior canal BPPV, and some patients become nauseated and require an antiemetic. Once the symptomatic ear has been identified, the mechanism and its different repositioning in the horizontal plane (“barbecue” repositioning) are explained to the patient (Figure 11). With the examiner seated at the head of the examination couch the patient is asked to rotate 360 degrees in four stages, a minute apart. At the third position the patient should be resting on the elbows with the neck flexed, so that the horizontal canal is vertical, which is where the particle will exit the canal if it has been successfully moved. The head roll test is repeated and, if negative, treatment ceases.

If on the head roll test the nystagmus is apogeotropic, the likely mechanism is cupulolithiasis in the undermost ear with the* least* nystagmus. The particle(s) could be on either side of the cupula. On the presumption it is on the canal side the standard direction rotation is carried out. If unsuccessful (likely vestibule side) rotation is done in the opposite direction. Additional headshaking or mastoid vibration can be used. Sometimes apogeotropic nystagmus reverses to geotropic, implying that an attached particle(s) has become free. If there is not a clear pattern, or if the patient becomes very nauseated, it is advisable to retest on another day. Central pathology must be kept in mind.

### 2.3. Superior Canal BPPV

On a Dix Hallpike test if there is downbeat nystagmus superior canal, BPPV is a possibility. If the cause is central, the nystagmus onset is immediate, and the patient does NOT experience vertigo. If it is BPPV, there will be a latency of onset and the patient MUST experience vertigo. Any torsional component may be imperceptible to the naked eye. Repeating the test with the head lower (“head-hanging” test) than usual may intensify the response. The particle may be in either ear but most likely in the uppermost ear. On these assumptions there are two simple “office” treatments.

**Figure 12:**
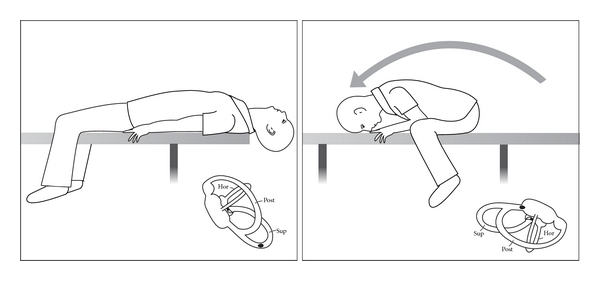
The Li manoeuvre for superior canal BPV in either ear (left ear).

The first is the Epley CRP performed with “head hanging” and commencing with the suspected ear *uppermost*. The second is the Li maneuver [65] where the patient is moved rapidly from a supine (midline) head-hanging position to a face-down position at the opposite end of the couch (Figure 12).

### 2.4. Recurrences, Failed Office Treatment, and Complications

Reported rates of spontaneous complete resolution of BPPV at a month range from 20% to 80%. The American Academy of Otolaryngology, Head and Neck Surgery Clinical Practice Guideline, Benign Paroxysmal Positional Vertigo recommends that physician retesting at one month after repositioning treatment should be the standard interval after treatment [58].

Patients treated for BPPV should be told that there is a likelihood of recurrences. Most trials involve a short follow-up period. In trials with longer followup the recurrence rate at one year is estimated at 15% [66] and 37%–50% at 5 years on the Kaplan-Meier curve [66, 67]. Posttraumatic BPPV may have a higher recurrence rate than spontaneous BPPV [68].

The most common “complication” of BPPV repositioning treatment is canal conversion. Considering the population age in which it is usually performed there is a surprising sparsity of literature on cervical spine and neurological complications [69].

### 2.5. Central Pathology

Central nervous system disorders can masquerade as BPPV, in particular intracranial tumours [52, 70, 71] and migraine. Nystagmus features which strongly suggest a neurological cause are downbeat nystagmus, direction-changing nystagmus without a change in head position, nausea with up or downbeating nystagmus, and preexisting and continuing nystagmus. The cardinal distinguishing feature from BPPV nystagmus is that the patient does NOT experience brief rotational vertigo. Therefore patients with such nystagmus or symptoms of BPPV not showing resolution after repositioning require neurological examination and MRI scanning of the brain and posterior fossa.

Migraine is now a well-recognised cause of recurrent vertigo [72]. During an episode positional testing can elicit upbeat torsional or horizontal nystagmus similar to BPPV [73, 74]. Features supporting migraine as a cause are headache, absence of brief acute vertigo induced by the Dix Hallpike test, disappearance of all nystagmus within days, and a recurrent pattern.

## 3. Future Directions

Currently Epley's canalith theory explains most of the features of BPPV: the latency and type of nystagmus, according to the involved canal, and the logic and efficacy of repositioning treatments. However, as for many other inner ear disorders, certain details of its pathophysiology, in particular spontaneous recovery, remain elusive, largely due to the inability to internally image the inner ear in enough detail and the prior reliance on histological techniques. Increased knowledge of human otoconial physiology and pathology will be important. 

As episodes of BPPV recover without treatment, it is reasonable to assume that otoconia exit a canal during normal head movement, particularly in horizontal canal and superior canal BPPV. It has been demonstrated that frog otoconia rapidly dissolve in endolymph with physiologic calcium levels, but more slowly if the calcium level is raised [75]. Therefore a major reason for spontaneous recovery is the ability of normal endolymph to dissolve otoconia if they do not return to the utricle. In mammals otoconia are calcite crystals of calcium carbonate. In rats scanning electron microscopy (EM) shows a progressive degeneration of otoconial structure in the oldest rats [76], a phenomenon consistent with the common age range in BPPV. In female rats made artificially oesteopenic/osteoporotic, scanning EM shows ultrastructural changes in otoliths [77] suggesting that there could be relationship between bone biochemistry and recurrent BPPV in older women. Seventy-five percent of thirty-two women aged been 50 and 85 years who had BPPV were shown to have osteopenia or osteoporosis compared with eighty-three healthy controls [78].

Measurement of vestibular-evoked myogenic potentials (VEMPs) is new electro-physiological technqiue for measuring saccule and otolith function. In some patients with vestibular neuritis absence of the cervical VEMPs(c-VEMP) on the side neurolabyrinthitis predicted the nondevelopment of BPPV which only occurred if the cVEMP was present. More recent studies on the “health” of the otolith organs using c-VEMPs [79, 80] have shown significantly large number of prolonged responses in patients with BPPV compared with healthy controls, suggesting neuronal degeneration of the saccule. In contrast newer ocular VEMPs (o-VEMP) also measure utricular function and are likely to be more relevant in establishing otolith dysfunction and recovery in BPPV. Distortion of the visual horizontal due to ocular torsion occurs in utricular dysfunction, as in vestibular neuritis. Measurement of the visual horizontal in BPPV patients was abnormal in 42% before-treatment, 15% after repositioning and in 8% two weeks later suggesting initial utricular dysfunction and its possible restoration from the return of otoliths [81]. Encouraging advances in imaging may eventually enable *in vivo *correlation. Three-dimensional T2-weighted 3-dimensional fast MRI imaging with steady-state acquisition sequences can now show some reliable detail of semicircular structure such as narrowed areas and “filling defects” [82]. In patients with “intractable” BPPV 89% had abnormal canals compared with healthy controls, but there was no correlation between the affected canal and nystagmus type. Subtle variations in canal diameter, length, and width may correlate with a predisposition to BPPV and to treatment failures.

Finally, the American Academy of Otolaryngology, Head and Neck Surgery Clinical Practice Guideline on BPPV [58] has recommended sixteen aspects meriting further research, including the true prevalence and burden of untreated BPPV in older adults, the natural history of untreated BPPV, agreed endpoints for clinical trials, and importantly the functional impact of BPPV on work safety and the rates of falls it may account for in the elderly.

## Supplementary Material

The supplementary videos show examples of BPPV from the posterior, horizontal and superior canals, and a Semont maneuver for right posterior canal BPPV.
